# Theoretical Studies on InGaAs/InAlAs SAGCM Avalanche Photodiodes

**DOI:** 10.1186/s11671-018-2559-5

**Published:** 2018-05-21

**Authors:** Siyu Cao, Yue Zhao, Sajid ur Rehman, Shuai Feng, Yuhua Zuo, Chuanbo Li, Lichun Zhang, Buwen Cheng, Qiming Wang

**Affiliations:** 1 State Key Laboratory on Integrated Optoelectronics, Institute of Semiconductors, Chinese Academy of Sciences, Beijing, 100083 China; 2 School of Science, Minzu University of China, Beijing, 100081 China; 3 College of Materials Science and Opto-Electronic Technology, University of Chinese Academy of Sciences, Beijing, 100049 China; 4 School of Physics and Optoelectronic Engineering, Ludong University, Yantai, 264025 China

**Keywords:** Avalanche photodiodes, Theoretical analysis, Simulation, Charge layer, Tunneling effect

## Abstract

In this paper, we provide a detailed insight on InGaAs/InAlAs separate absorption, grading, charge, and multiplication avalanche photodiodes (SAGCM APDs) and a theoretical model of APDs is built. Through theoretical analysis and two-dimensional (2D) simulation, the influence of charge layer and tunneling effect on the APDs is fully understood. The design of charge layer (including doping level and thickness) can be calculated by our predictive model for different multiplication thickness. We find that as the thickness of charge layer increases, the suitable doping level range in charge layer decreases. Compared to thinner charge layer, performance of APD varies significantly via several percent deviations of doping concentrations in thicker charge layer. Moreover, the generation rate (*G*_*btt*_) of band-to-band tunnel is calculated, and the influence of tunneling effect on avalanche field was analyzed. We confirm that avalanche field and multiplication factor (*M*_*n*_) in multiplication will decrease by the tunneling effect. The theoretical model and analysis are based on InGaAs/InAlAs APD; however, they are applicable to other APD material systems as well.

## Background

In_0.53_Ga_0.47_As (referred to hereafter as InGaAs) avalanche photodiodes (APDs) are the most important photodetectors for short-wave infrared detection. They are significant in traditional fields, such as optical fiber communication, reconnaissance applications, and remote sensing. InP and In_0.52_Al_0.48_As (referred to hereafter as InAlAs) have the same lattice spacing with InGaAs and great avalanche breakdown characteristics; therefore, they are the suitable multiplication layer materials of InGaAs APDs in the traditional applications. In recent years, due to the quick development of single-photon detection in quantum key distribution [1], time-resolved spectroscopy [2], optical VLSI circuit inspection [3], and 3D laser ranging [4], APDs as the key component in these applications have attracted increasing attention [5, 6]. Pellegrini et al. described the design, fabrication, and performance of planar-geometry InGaAs/InP devices which were developed for single-photon detection with the single-photon detection efficiency (SPDE) 10% at 1550 nm (200 K) [7]. Tosi et al. presented the design criteria of a novel InGaAs/InP single-photon avalanche photodiode (SPAD) with high SPDE (30%, 225 K), low noise, and low timing jitter [8]. In simulation, a device model based on experimental data was built to predict dark count rate (DCR) and SPDE of InGaAsP/InP SPADs in [9], and an integrated simulation platform that can evaluate the decoy-state quantum key distribution performance of InGaAs/InP SPADs was built in [10]. Acerbi et al. presented design criteria for InGaAs/InP single-photon APDs with a custom SPAD simulator [11]. For InGaAs/InAlAs APDs, a mesa structure SPAD InGaAs/InAlAs was demonstrated to achieve the SPDE of 21% (260 K); however, high DCR was observed and was attributed to excessive tunneling current [12]. Then, [13] used a thick InAlAs avalanche layer in InGaAs/InAlAs APDs to improve the SPDE (26%, 210 K) and decrease the DCR (1 × 10^8^ Hz). In simulation of InAlAs-based APDs, a device model that based on the Monte Carlo method was established to study the basic characterization of InGaAs/InAlAs APDs in [14], and the influence of charge layer and multiplication layer on punchthrough voltage and the breakdown voltage were studied with steady-state 2D numerical simulations in [15].

Compared to InAlAs-based APDs, researches of InP-based APDs are more comprehensive and in depth in theory and simulation. However, InAlAs-based APD is increasingly used in place of InP as it has a larger band gap that can improve the breakdown characteristics both in the APDs and SPADs [16]. The ionization coefficient ratio of electron (α) to hole (β) in InAlAs is larger compared to InP, and, hence, it has low excess noise factor and high gain-bandwidth product. Moreover, InAlAs exhibits a large increase in breakdown probability with overbias ratio, making InAlAs SPADs have lower DCR [17]. Some important properties and conclusions regarding InAlAs-based APDs were obtained from previous studies, such as the low excess noise can be achieved in InAlAs structures with both thick and thin avalanche regions [18]. The tunneling threshold electric field in the absorption (InGaAs) is 1.8 × 10^5^ V/cm, and the tunneling current becomes the dominant component of the dark current in the high field [14]. A vertical-illumination structure has larger optical tolerance, but it has a more serious tradeoff between the carrier transit time and responsivity [19]. Moreover, theoretical model, structure (thickness and doping), electric field, and other InAlAs-based APD parameters have been studied in [20–22]. However, these studies only focused on influences of common APD structure parameters, such as the absorption layer thickness, multiplication thickness, and charge layer doping level. The relationship between the structure parameters and performance of the InAlAs-based APD has not yet been fully understood and optimized.

In this paper, a theoretical study and numerical simulation analysis based on the vertical structure of InGaAs/InAlAs for 1.55-μm wavelength detection were investigated. We built a theoretical model to study the influence of structure parameters on device and detailed relationship of each layer in APDs. The design of the charge layer with different multiplication thickness, influence of the thickness on the doping level in charge layer, and the tunneling effect on the avalanche field in the multiplication were analyzed and simulated.

## Methods

In this section, a mathematical relationship between the device parameters and electric field distribution in the device was built, which was applied to analyze the charge layer and the tunneling effect. Concurrently, a simulation model that included simulation structure, material parameters, and basic physical models was built. The theoretical analysis model and simulation model was based on the vertical structure of SAGCM InGaAs/InAlAs APD.

### Theoretical Model and Analysis of Charge Layer

**Fig. 1:**
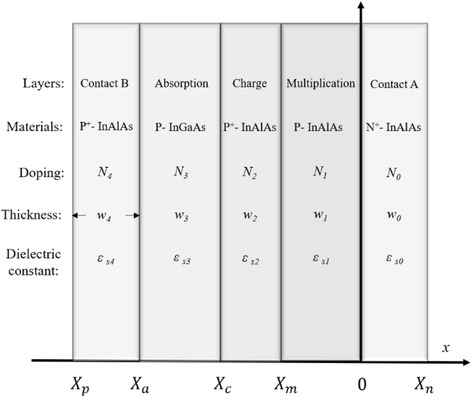
The simplified mathematical coordinate system of SACM InGaAs/InAlAs APD. Presents the simplified structures of an APD that is used to build a theoretical model. The simplified mathematical coordinate system of the APD that includes basic structure parameters (materials, thickness, doping, and dielectric constant)

Device parameters, such as doping level, thickness, materials, and structure, were used to build the mathematical model for calculating the electric field distribution in APD. The basic physical theories that include Poisson equation, depletion-layer model, and PN junction model of semiconductor device can be found in chapters 1, 2, and 4 in [23] and [24]. The junction multiplication factor equation can be found in [25], and material parameters of semiconductor are from [26]. The presented model adopts Poisson equation, tunneling current density equation, depletion-layer model, junction theory model, and the local model of avalanche gain. The simplified mathematical coordinate system of the APD that includes basic structure parameters (materials, thickness, doping, and dielectric constant) is presented in Fig. 1. It is a simplified SACM APD structure that ignores grading layer. The materials of the contact layer, charge layer, and multiplication layer are InAlAs, and the absorption layer is InGaAs. The junctions of layers are separated by *X*_*n*_, 0, *X*_*m*_, *X*_*c*_, and *X*_*a*_ and *X*_*p*_ by the *x* coordinate. Doping levels are expressed by *N*_*0*_, *N*_*1*_, *N*_*2*_, *N*_*3*_, and *N*_*4*_, the layer thicknesses are expressed by *w*_*0*_, *w*_*1*_, *w*_*2*_, *w*_*3*_, and *w*_*4*_, and dielectric constants are expressed by *ε*_*s0*_, *ε*_*s1*_, *ε*_*s2*_, *ε*_*s3*_, and *ε*_*s4*_ of contact A, multiplication, charge, absorption, and contact B, respectively.

Equation 1 is the Poisson equation, which can solve the electric potential distribution using the charge density *ρ*. In this equation, *ρ* is equal to dopant ion *N* in the depletion-layer model, *w* is equal to the thickness of depletion layer, and *ε* is the dielectric constant of the material. In the common PN junction electric field distribution model, *ρ* is a variable that depends on the depletion-layer thickness *w* and dopant ion *N*. In this model, it changes after considering the tunneling effect. However, before considering the tunneling effect, we first built the electric field distribution using a common method.

1 $$ \frac{d\xi}{d x}=\frac{\rho }{\varepsilon }=\frac{q\times N}{\varepsilon } $$

By solving the Poisson equation with the device parameters, the mathematical expression of the max electric field is obtained. This expression is determined by the penetration thickness variation in the depletion layer shown in Formulas 2 and 3. In this expression, the parameters that include doping levels (*N*), thicknesses of depletion layer (*w*), and dielectric constants (*ε)* of different layers can be found in Fig. 1.

2 $$ {\xi}_{\max (w)}={\sum}_{k=1}^4\left(-\frac{q\times {N}_k\times {w}_k}{\varepsilon_{sk}}\right) $$

3 $$ {\xi}_{\max (w)}=\frac{q\times {N}_0\times {w}_0}{\varepsilon_{s0}} $$

Then, the electric field distribution can be derived in all points using Formulas 4 and 5. The boundary condition ignores the built-in potential *V*_br_ in Formula 6; therefore, the mathematical relationship between depletion layer thickness and bias voltage can be calculated.

4 $$ {\xi}_{\left(x,w\right)}={\xi}_{\max (w)}+{\sum}_{k=1}^4\left(\frac{q\times {N}_k\times \left|x\right|}{\varepsilon_{sk}}\right)\left({X}_p<x<0\right) $$

5 $$ {\xi}_{\left(x,w\right)}={\xi}_{\max (w)}-\frac{q\times {N}_0\times x}{\varepsilon_{s0}}\left(0<x<{X}_n\right) $$

6 $$ {v}_{\mathrm{bi}\mathrm{as}}+{V}_{\mathrm{bi}}=-{\int}_{-{w}_0}^{w_1+{w}_2+{w}_3+{w}_4}\xi \left(x,w\right) dx $$

Finally, the mathematical relationship between electric field distribution and bias voltage in the device is obtained using Formulas 7–11:

7 $$ \xi \left(x,{V}_{\mathrm{bias}}\right)={\xi}_{\max \left({V}_{\mathrm{bias}}\right)}+\frac{q\times {N}_1\times \left|x\right|}{\varepsilon_{s1}}\left(0\ge x\ge {X}_m\right) $$

8 $$ \xi \left(x,{V}_{\mathrm{bias}}\right)={\xi}_{\max \left({V}_{\mathrm{bias}}\right)}+\frac{q\times {N}_1\times {w}_1}{\varepsilon_{s1}}+\frac{q\times {N}_2\times \left|x-{X}_m\right|}{\varepsilon_{s2}}\left({X}_m\ge x\ge {X}_c\right) $$

9 $$ \xi \left(x,{V}_{\mathrm{bias}}\right)={\xi}_{\max \left({V}_{\mathrm{bias}}\right)}+\frac{q\times {N}_1\times {w}_1}{\varepsilon_{s1}}+\frac{q\times {N}_2\times {w}_2}{\varepsilon_{s2}}+\frac{q\times {N}_3\times \left|x-{X}_c\right|}{\varepsilon_{s3}}\left({X}_c\ge x\ge {X}_a\right) $$

10 $$ \xi \left(x,{V}_{\mathrm{bias}}\right)={\xi}_{\max \left({V}_{\mathrm{bias}}\right)}+\frac{q\times {N}_1\times {w}_1}{\varepsilon_{s1}}+\frac{q\times {N}_2\times {w}_2}{\varepsilon_{s2}}+\frac{q\times {N}_3\times {w}_3}{\varepsilon_{s3}}+\frac{q\times {N}_4\times \left|x-{X}_a\right|}{\varepsilon_{s4}}\left({X}_a\ge x\ge {X}_p\right) $$

11 $$ \xi \left(x,{V}_{\mathrm{bias}}\right)={\xi}_{\max \left({V}_{\mathrm{bias}}\right)}-\frac{q\times {N}_0\times x}{\varepsilon_{s0}}\left(0\le x\le {X}_n\right) $$

From the model, once the boundary of the depletion layer reaches the contact region, Formulas 7–11 can be used to analyze the electric field in each layer. In the practical APD, the absorption and multiplication layers are unintentionally doped in intrinsic layers. *N*_*3*_ and *N*_*1*_ are less than *N*_*2*_. Thus, Formula 9 is approximately equal to Formula 12. It is the reason that charge layer can control the electric field distribution in the device.

12 $$ {\displaystyle \begin{array}{l}\xi \left(x,{V}_{\mathrm{bias}}\right)={\xi}_{\max \left({V}_{\mathrm{bias}}\right)}+\frac{q\times {N}_1\times {w}_1}{\varepsilon_{s1}}+\frac{q\times {N}_2\times {w}_2}{\varepsilon_{s2}}+\frac{q\times {N}_3\times \left|x-{X}_c\right|}{\varepsilon_{s3}}\\ {}\kern4em \approx {\xi}_{\max \left({V}_{\mathrm{bias}}\right)}+\frac{q\times {N}_2\times {w}_2}{\varepsilon_{s2}}\left({X}_{\mathrm{c}}\ge x\ge {X}_a\right)\end{array}} $$

**Fig. 2:**
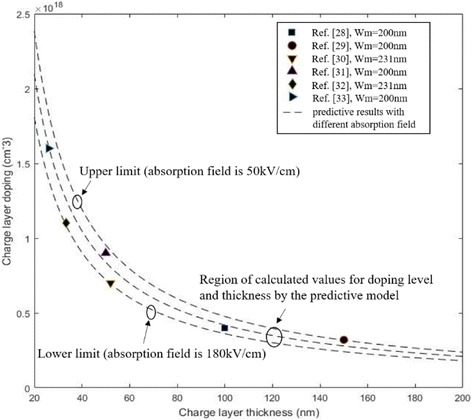
Comparison of theoretical results and experiment data from various reports (*w*_*m*_ = 200 nm). Closed symbols: the doping level and thickness of charge layer with multiplication thickness of 200 nm (black square, black circle, black triangle, black right-pointing triangle) and 231 nm (black diamond, black down-pointing triangle) in the references. Presents the calculated values of charge layer (doping level and thickness) by Formula 8 (the absorption field is 50–180 kV/cm). When the absorption field is 50 kV/cm, the upper limit of the doping level in the charge layer can be obtained. When the absorption field is 180 kV/cm, the lower limit of the doping level in the charge layer can be obtained. We compare the theoretical results and experiment data from various reports. The region of theoretical values is in good agreement with the experimental data. Dashed lines the calculated values of doping level and thickness by the formula

In Formula 8, the electric field difference between multiplication and absorption is determined using the product of *N*_*2*_ and *w*_*2*_. *N*_*2*_ is the doping level in the charge layer and *w*_*2*_ is the charge layer thickness. For a suitable electric field distribution in InGaAs/InAlAs APD, the electric field in the absorption layer (InGaAs) should be within the interval values of 50–180 kV/cm that ensure enough velocity for the photo-induced carriers and avoid the tunneling effect in the absorption layer [10]. That is, the avalanche field in multiplication should decrease to 50–180 kV/cm in absorption by the charge layer. Thus, we can use Formula 8 to find optimal calculated doping level and thicknesses of charge layer. When the multiplication layer is 200 nm (the avalanche field *E* in the multiplication is 6.7 × 10^5^ V/cm while the multiplication layer is 200 nm [27]); the calculated values of doping level and thickness in the charge layer are compared with results from [28–33] in Fig. 2. The region of theoretical values is in good agreement with the experimental data. This result proves that Formula 8 can be used to predict the doping level with different thicknesses in the charge layer when the multiplication thickness is certain.

**Fig. 3:**
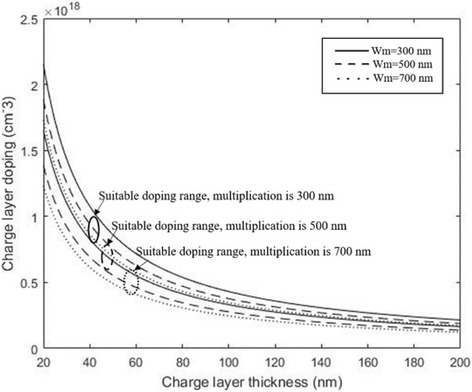
The optimal doping level and thickness of charge layer for different multiplication layer. Solid line: *w*_*m*_ = 300 nm. Dashed line: *w*_*m*_ = 500 nm. Dot line: *w*_*m*_ = 700 nm. Presents the calculated values of charge layer (doping level and thickness) by the formula while the field of absorption layer is suitable. The thicknesses of the multiplication layer are 300, 500, and 700 nm. When the thickness of the multiplication layer is certain, we can use the formula to find the optimal doping level and thickness of charge layer

We calculate the optimal doping level for different thicknesses of the charge layer with the multiplication layer of 300, 500, and 700 nm, and the results are presented in Fig. 3. This result illustrates that the tolerance in the doping level in charge layer is related to its thickness and the range of doping level decreases with the thickness increase in charge layer. That is, if we apply a thick charge region, only a small range of doping level in the charge layer would exist to satisfy the optimal electrical filed. As a result, the performance of APD varies significantly via several percent deviations of doping concentrations in the thicker charge layer. In the “Results and Discussion” section, the practical structures of APDs were simulated to study and verify the theoretical analysis, which includes influence of charge layer thickness on doping level range in the charge layer and the variety of performance for different charge layer thickness in APDs.

### Theoretical Model with Consideration of Tunneling

**Fig. 4:**
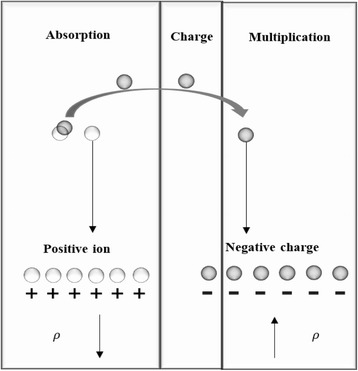
Tunneling process and charge density change in the multiplication and absorption layers. Presents a schematic diagram of tunneling process in the device. If a sufficiently high electric field exists within the absorption layer, the local band bending may be sufficient to allow electrons to tunnel. When the absorption layer has a breakdown tunneling, the positive charge in absorption increases and the negative charge in the multiplication and charge layers increases. Thus, *ρ* is not equal to the dopant ion charge density in the depletion layer while the tunneling effect appears

The above analysis model is about electric field distribution in the device and based on the premise that *ρ* is the dopant ion in the depletion layer. If a sufficiently high electric field exists within the absorption layer, the local band bending may be sufficient to allow electrons to tunnel [34]. Therefore, electron tunneling can occur. From the tunneling schematic diagram in Fig. 4, when the absorption layer has a breakdown tunneling, the tunneling effect changes the charge density *ρ*, the positive charge in absorption increases, and the negative charge in the multiplication and charge layers increases. Thus, *ρ* is not equal to the dopant ion charge density in the depletion layer while the tunneling effect appears. The formulas that were discussed earlier will change after considering the tunneling effect.

The generation rate *G*_*bbt*_ of band-to-band tunnel is described in Formula 13 [35, 36].

13 $$ {G}_{bbt}={\left(\frac{2{m}^{\ast }}{E_g}\right)}^{1/2}\frac{q^2{E_p}^{\gamma }}{{\left(2\pi \right)}^3{\hbar}^2}\exp \left(\frac{-\pi }{4{q\mathit{\hbar E}}_p}{\left(2{m}^{\ast}\times {E_g}^3\right)}^{\raisebox{1ex}{$1$}\!\left/ \!\raisebox{-1ex}{$2$}\right.}\right)=A\times {E_p}^{\gamma}\times \exp \left(-\frac{B}{E_p}\right) $$

**Fig. 5:**
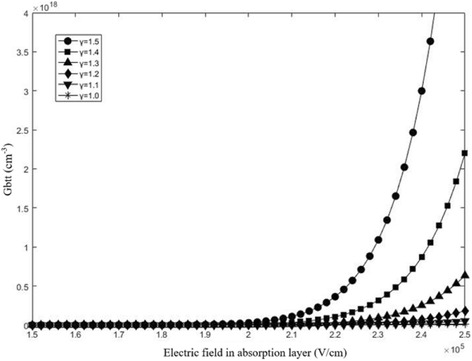
The *G*_*btt*_ for different fields in absorption layer with different *γ*. The values of *γ* is 1.0 (black star), 1.1 (black down-pointing triangle), 1.2 (black diamond), 1.3 (black triangle), 1.4 (black square), 1.5(black circle). Presents the calculated results of *G*_*btt*_ by Formula 13. When the field of absorption exceeds 19 kV/cm, *G*_*bbt*_ gradually increases. It also can be found that *G*_*bbt*_ adapts the same order of magnitude for the charge layer doping level while *γ* is restricted to 1~1.5

In Formula 13, *E*_*g*_ is the energy band gap of InGaAs, m* (equal to 0.04 *m*_*e*_) is the effective reduced mass, *E*_*p*_ is the breakdown electric field in the absorption layer, and *γ* is a user-definable parameter that is usually restricted to 1~2. The *A* and *B* are the characterization parameters. We calculate the *G*_*bbt*_ with different *γ*, and the results are shown in Fig. 5. It can be found that *G*_*bbt*_ adapts the same order of magnitude for the charge layer doping level while *γ* is restricted to 1~1.5.

As a result, charge density *ρ* is a variable and determined by the tunneling effect and the dopant ion in the absorption tunnel. On this occasion, Formula 1 will be changed to Formula 14 and the electric field in the multiplication layer will be described by Formula 15. *w*_tunnel_ is the effective depletion layer of the tunneling process [35]. Thus, the changing of avalanche field can be described by Formula 16, and the avalanche field will decrease in the multiplication with the tunneling effect.

14 $$ \frac{d\xi}{d x}=\frac{\rho }{\varepsilon }=\frac{q\times \left(N+{G}_{btt}\right)}{\varepsilon },{E}_p>1.8\times {10}^5V/ cm $$

15 $$ \xi \left(x,{V}_{\mathrm{bias}}\right)={\xi}_{\max \left({V}_{\mathrm{bias}}\right)}+\frac{q\times \left({N}_1\times \left|x\right|+{G}_{bbt}\times {w}_{\mathrm{tunnel}}\right)}{\varepsilon_{s1}}\left(0\ge x\ge {X}_m\right) $$

16 $$ \delta \xi \left(x,{V}_{\mathrm{bias}}\right)=\delta E=\frac{q\times {G}_{btt}\times {w}_{\mathrm{tunnel}}}{\varepsilon_{\mathrm{s}3}} $$

The electron and hole ionization coefficients are described by Formulas 17 and 18 in [18]. *E* is the avalanche field in multiplication.

17 $$ \alpha ={a}_n{e}^{\raisebox{1ex}{$-{b}_n$}\!\left/ \!\raisebox{-1ex}{$E$}\right.} $$

18 $$ \beta ={a}_p{e}^{\raisebox{1ex}{$-{b}_p$}\!\left/ \!\raisebox{-1ex}{$E$}\right.} $$

The effect of carrier avalanche is accounted by the impact ionization model. Considering the extremely low carrier density of the multiplication layer compared to charge layer, it is reasonable to assume that the electric field is uniform throughout the multiplication layer. Therefore, the multiplication factor (*M*_*n*_) can be expressed as the following Eq. 19. Here, *w*_*m*_ is the multiplication layer thickness and *k* is the impact ionization coefficient ratio defined by *α/β*. Since *k* varies very slowly with the electric field, *k* is approximately constant for a slight variation of *w*_*m*_ [37].

19 $$ {M}_n=\frac{k-1}{k\times {e}^{-\alpha \left(1-\raisebox{1ex}{$1$}\!\left/ \!\raisebox{-1ex}{$k$}\right.\right){w}_m}-1} $$

Assuming constant *w*_*m*_ and bias voltage, differentiation of *M*_*n*_ with respect to electron ionization coefficients is in Formulas 20 and 21.

20 $$ \delta {M}_n\left|{}_{w= const\&V= const}\right.={M_n}^2{e}^{-\alpha \left(1-\raisebox{1ex}{$1$}\!\left/ \!\raisebox{-1ex}{$k$}\right.\right){w}_m}\times {w}_m\delta \alpha $$

21 $$ \delta \alpha =\frac{\delta \alpha}{\delta E}={\alpha}_n{b}_n{e}^{\frac{-{b}_n}{E}}\frac{1}{E^2} $$

**Fig. 6:**
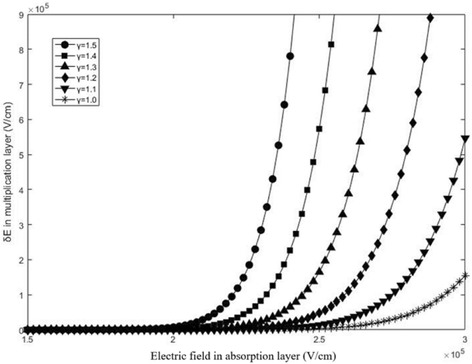
The *δE* for different fields in the absorption layer with different *γ*. The values of *γ* is 1.0 (black star), 1.1 (black down-pointing triangle), 1.2 (black diamond), 1.3 (black triangle), 1.4 (black square), 1.5(black circle). Presents the calculated results of *δE* by Formula 16. When the field of absorption exceeds 19 kV/cm, *δE* gradually increases. It also can be found that *δE* adapts the same order of magnitude for the avalanche field in the multiplication. Thus, the tunneling effect has an influence on the avalanche field with the tunneling effect

In Formulas 20 and 21, *δα/δE* is positive. It is assumed that 20% of a total depletion absorption layer is *w*_tunnel_ and the absorption layer is 400 nm thick. By solving Formula 16, the relationship between the *δE* and the absorption field with different *γ* is presented in Fig. 6. It can be found that *δE* adapts the same order of magnitude for the avalanche field in the multiplication. Thus, the tunneling effect has an influence on the avalanche field and the *M*_*n*_ will decrease with the tunneling effect. In the analysis, we assumed that the negative charge is non-multiplied in the multiplication and the model will be more rigorous after taking it into consideration. To verify and analyze the influence of tunneling effect on practical structure of APDs, we simulated the relationship between the tunneling effect and multiplication avalanche field in details in the “Results and Discussion” section.

### Structure and Simulation Model

A semiconductor device simulation of TCAD was used for simulation and analysis. This simulation engine defines physical models in simulation, and the results have a physical meaning [20]. The basic physical models were presented as follows. The drift-diffusion models, including the Poisson and carrier continuity equations, were used to simulate the electric field distribution and diffusion current I_DIFF_. Band-to-band tunneling model was used for the band-to-band tunneling current I_B2B_, and the trap-assisted tunneling model was used for trap-assisted tunneling current I_TAT_. The generation-recombination current I_GR_ was described by the Shockley–Read–Hall recombination model, and the Auger recombination current I_AUGER_ was described by the Auger recombination model. The dark current was described clearly by those mechanisms [38]. Avalanche multiplication was described by the Selberherr impact ionization model. Other basic models, including the Fermi-Dirac carrier statistics, carrier concentration-dependent, low field mobility, velocity saturation, and ray-tracing methods, were used for the simulation model, and a rigorous simulation model was built.

**Fig. 7:**
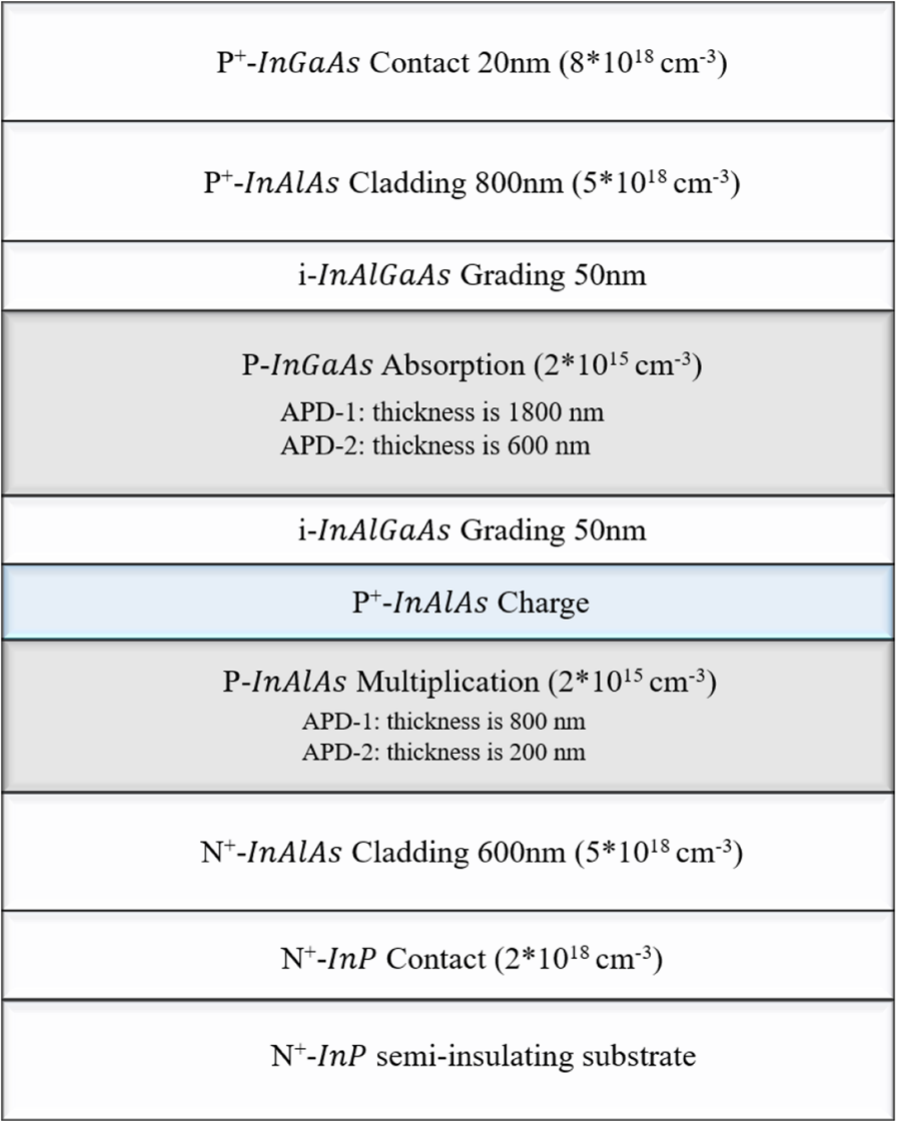
Simulation structure and parameters of the APD. Presents the schematic cross-section of the top-illuminated SAGCM InGaAs/InAlAs APD-1 and APD-2. It includes structure, materials, doping, and thickness

Device structures in the simulation were similar to the experimental structures in [13]. The schematic cross-section of the top-illuminated SAGCM InGaAs/InAlAs APD is shown in Fig. 7. The structures from top to bottom are sequentially named as InGaAs contact layer, InAlAs cladding layer, InAlGaAs grading layer, InGaAs absorption layer, InAlGaAs grading layer, InAlAs charge layer, InAlAs multiplication layer, InAlAs cladding layer, InP contact layer, and InP substrate. The thickness and doping of each layer are also presented in Fig. 7. To avoid the influence of thickness on simulation results, we choose two simulation structures. One simulation structure is named as APD-1 (multiplication and absorption layers are 800 and 1800 nm, respectively), and the other simulation structure is named as APD-2 (multiplication and absorption layers are 200 and 600 nm, respectively).

**Fig. 8:**
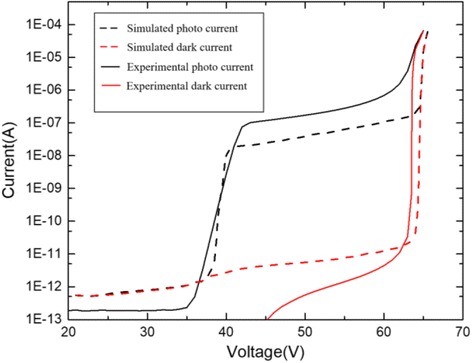
Simulation results compared with the experiment results (photocurrent and dark current). Black dashed line: simulated photo current. Red dashed line: simulated dark current. Black solid line: experimental photo current. Red solid line: experimental dark current. Presents the comparison of the simulation results and experiment results. The simulation model uses the same parameters from the experiment in the reference

To test the simulation model, the experiment data in [13] were compared with the simulation results. In this simulation, we used the same structure in the reference, and the current-voltage characteristics of the device were given. Figure 8 shows our simulation results and the experiment results in the reference. They have the similar punch-through voltage *V*_pt_ and breakdown voltage *V*_br_. Moreover, the simulation and experiment results correspond well. Therefore, the model in our simulation is accurate. The parameters mentioned above are listed in Table 1.

**Table 1:** Material parameters used for InGaAs/InAlAs APD simulation [18, 26]

Parameters/InAlAs	Units	Electron	Hole
Impact coefficient a	cm^−1^	2.1 × 10^6^	2.4 × 10^6^
Impact coefficient b	V/cm	1.62 × 10^6^	1.86 × 10^6^
Effective threshold energy	ev	3.2	3.5
SRH lifetime	s	1 × 10^−6^	1 × 10^−6^
Energy band gap	ev	1.46	
Parameters/InGaAs	Units	Electron	Hole
BBT coefficient A	1/V cm	7.2 × 10^19^	
BBT coefficient B	V/cm	5.2 × 10^6^	
Energy band gap	ev	0.75	

## Results and Discussion

In this section, the theoretical analysis and conclusions were studied by simulation in details. First, the influence of charge layer thickness on doping level tolerance in charge layer was studied in the “Influence of Charge Layer Thickness” section. Then, relationship between the tunneling effect and multiplication avalanche field was analyzed and verified in the “Tunneling Effect on the Electric Field Distribution” section.

### Influence of Charge Layer Thickness

From [14], a suitable field distribution in InGaAs/InAlAs APD should comply with those rules. The guarantee *V*_pt_ < *V*_br_ and *V*_br_ − *V*_pt_ should have a safety margin for processing variations in temperature fluctuations and operation range. In the absorption layer, the electric field should be larger than 50–100 kV/cm to ensure enough velocity for the photo-induced carriers. Concurrently, the electric field must be less than 180 kV/cm to avoid the tunneling effect in the absorption layer. Electric field distribution greatly influences the device performance. The choice of electric field in the absorption layer has a balancing of the tradeoff between small transit time, dark current, and high responsivity for the practical requirement.

**Fig. 9:**
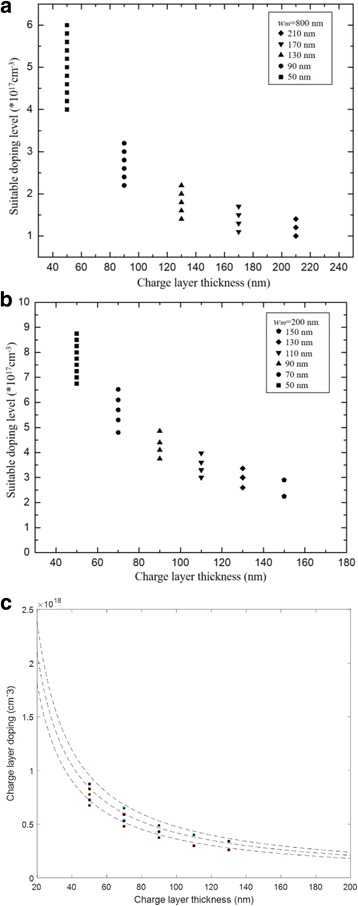
**a** Relationship between suitable doping level and thickness of charge layer (APD-1). The thickness of charge layer is 50 nm (black square), 90 nm (black circle), 130 nm (black triangle), 170 nm (black down-pointing triangle), 210 nm (black diamond). **a** presents the suitable doping level region for different thickness of charge layer. As the charge layer thickness increases, the suitable doping level range in the charge layer decreases. A relatively large doping level range exists in the thin charge layer, and under this doping level range, the device will have a suitable electric field distribution. Apparently, the doping level range is determined by charge layer thickness. **b** Relationship between suitable doping level and thickness of charge layer (APD-2). The thickness of charge layer is 50 nm (black square), 70 nm (black circle), 90 nm (black triangle), 110 nm (black down-pointing triangle), 130 nm (black diamond), and 150 nm (black pentagon). The figure description of **b** is similar to **a**. **c** Comparison of calculated results in Fig. 2 and simulated results in Fig. 9b. Dashed line: calculated results. Closed symbols: simulated results (black square). **c** presents the comparison of calculated results in Fig. 2 and simulated results in Fig. 9b. The calculated results and simulated results correspond well

In the simulation, we used the structure of APD-1 (multiplication is 800 nm thick) and adjusted the charge layer thickness from 50 to 210 nm to study the influence of charge layer thickness on doping level range and verify the theoretical conclusions in analytical model. In the simulation, we selected different doping level ranges in the charge layer so that the electric field distribution complies with the rules. The simulation results on the relationship between thickness and doping level range in the charge layer are presented in Fig. 9a. As the charge layer thickness increases, the suitable doping level range in charge layer decreases. A relatively large doping level range exists in the thin charge layer, and under this doping level range, the device will have a suitable electric field distribution. Apparently, the doping level range is determined by charge layer thickness. The simulation result of APD-2 (with a thickness of multiplication of 200 nm) is presented in Fig. 9b, which has a similar result. Moreover, it can be found that the calculated results of Fig. 2 and simulation results of Fig. 9b match well as shown in Fig. 9c. The small difference between the calculated results and simulation results is caused by the different values of avalanche field in the simulation and calculation. The avalanche field in simulation engine is used 6.4 × 10^5^ V/cm, while in the calculation, we use the value of 6.7 × 10^5^ V/cm from [27].

**Fig. 10:**
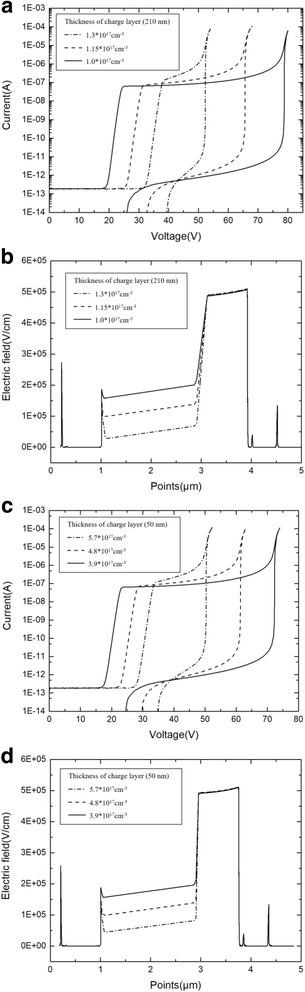
**a** Photocurrent and dark current with different doping level (thickness of charge layer is 210 nm). Solid line: doping level in the charge layer is 1.3 × 10^17^ cm^−3^. Dashed line: doping level in charge layer is 1.15 × 10^17^ cm^−3^. Dashed dot line: doping level in charge layer is 1.0 × 10^17^ cm^−3^. **a** Presents the simulation results of currents with different doping level. The device with a charge layer thickness of 210 nm only has a relatively narrow and suitable doping level. A minimal change in the doping level has greatly influence the punch-through voltage, breakdown voltage, and current-voltage characteristic. **b** Avalanche field with different doping level (thickness of charge layer is 210 nm). Solid line: doping level in charge layer is 1.3 × 10^17^ cm^−3^. Dashed line: doping level in charge layer is 1.15 × 10^17^ cm^−3^. Dashed dot line: doping level in charge layer is 1.0 × 10^17^ cm^−3^. **b** Presents the simulation results of fields with different doping level. The device with a charge layer thickness of 210 nm only has a relatively narrow and suitable doping level. A minimal change in the doping level has greatly influenced the electric field distribution. **c** Photocurrent and dark current with different doping level (thickness of charge layer is 50 nm). Solid line: doping level in charge layer is 5.7 × 10^17^ cm^−3^. Dashed line: doping level in charge layer is 4.8 × 10^17^ cm^−3^. Dashed dot line: doping level in charge layer is 3.9 × 10^17^ cm^−3^. **c** Presents the simulation results of currents with different doping level. The device with a charge layer thickness of 50 nm has a relatively wide and suitable doping level. A minimal change in the doping level has a small influence on the current-voltage characteristic. **d** Avalanche field with different doping level (thickness of charge layer is 50 nm). Solid line: doping level in charge layer is 5.7 × 10^17^ cm^−3^. Dashed line: doping level in charge layer is 4.8 × 10^17^ cm^−3^. Dashed dot line: doping level in charge layer is 3.9 × 10^17^ cm^−3^. **d** Presents the simulation results of fields with different doping level. The device with a charge layer thickness of 50 nm only has a relatively wide and suitable doping level. A minimal change in the doping level has a small influence on the electric field distribution

The charge layer thicknesses of 210 and 50 nm (APD-1) were selected to show the simulation details and the influence of doping level on the electric field distribution. Figure 10a, c shows the current simulation results of different doping levels in thicknesses of 210 and 50 nm, respectively. Figure 10b, d shows the electric field distribution simulation results using the same structure. The simulation results show that thicknesses of 210 and 50 nm have doping level ranges of 1.0 × 10^17^–1.3 × 10^17^ cm^−3^ and 3.9 × 10^17^–5.7 × 10^17^ cm^−3^, respectively.

Clearly, the device with a charge layer thickness of 210 nm only has a relatively narrow and suitable doping level. A minimal change in the doping level has greatly influence the current-voltage characteristic and electric field distribution. As a result, the performance of APD varies significantly via several percent deviations of doping concentrations in the thicker charge layer. This conclusion is the same as the theoretical analysis. Concurrently, when designing APD structures, choosing a thin charge layer will give a high level of doping tolerance, as well as confer APD with good controllability.

**Fig. 11:**
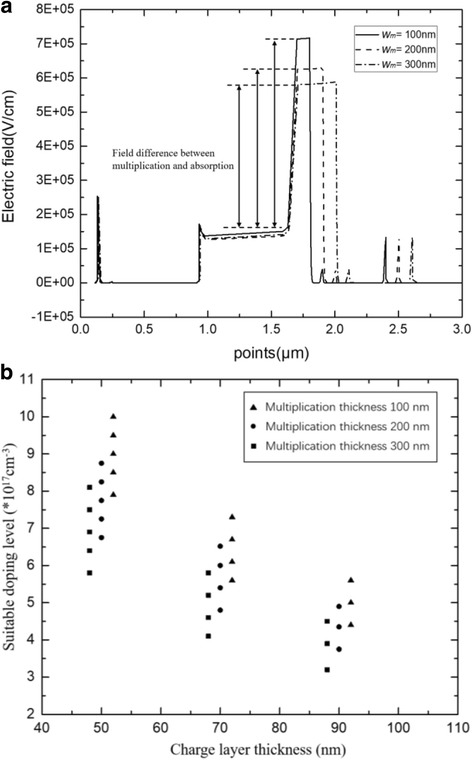
**a** Avalanche breakdown electric field with different multiplication thicknesses. Solid line: *w*_*m*_ = 100 nm. Dashed line: *w*_*m*_ = 200 nm. Dashed dot line: *w*_*m*_ = 300 nm. **a** Presents the simulation results of electric field distribution with different *w*_*m*_. As the *w*_*m*_ decreases, the avalanche field in the multiplication increase. **b** Relationship between multiplication thickness and charge layer. The thickness of multiplication is 300 nm (black square), 200 nm (black circle), 100 nm (black triangle). **b** Presents the relationship between multiplication thickness and charge layer. A thin multiplication layer needs a high product of the charge layer doping level and thickness to reduce the high avalanche field

Finally, the relationship between charge layer and multiplication thickness was simulated. Figure 11a presents the avalanche field with multiplication region thicknesses of 100, 200, and 300 nm in the APD-2 structure (with a charge layer thickness of 70 nm). Figure 11b presents the charge layer doping range with different multiplication thicknesses at the suitable electric field distribution condition. The charge layer thicknesses are 50, 70, and 90 nm. Clearly, a high avalanche field exists in the thin multiplication layer. As the multiplication region thickness decreases, the electric field difference between multiplication and absorption layers increases. As a result, a thin multiplication layer needs a high product of the charge layer doping level and thickness to reduce the high avalanche field.

### Tunneling Effect on the Electric Field Distribution

**Fig. 12:**
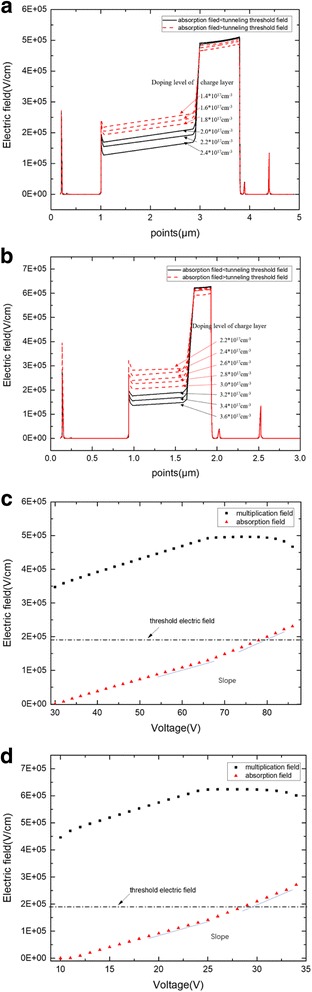
**a** Avalanche breakdown electric field with different doping levels (APD-1). Thickness of charge layer is 90 nm. Red dashed lines: the field of absorption is larger than the tunneling threshold field. Black solid lines: the field of absorption is less than the tunneling threshold field. **a** Presents the simulation results of electric field distribution with different doping level while avalanche breakdown. When doping level of charge layer exceeds 2.0 × 10^17^ cm^−3^, the field in the absorption layer becomes lower than the tunneling threshold field, and the avalanche field in the multiplication layer reaches the same value with different doping level. However, when the doping level is less than 2.0 × 10^17^ cm^−3^, the field in the absorption layer exceeds the tunneling threshold field, and the avalanche field in the multiplication layer decreases with the decrease of the doping level. Thus, if the electric field in the absorption layer exceeds the tunneling threshold value at or over the breakdown voltage, then the breakdown electric field in the multiplication will decrease. Thus, the electric field in the absorption should be less than the tunneling threshold value to maintain the high field in the multiplication layer when the device avalanche breaks down. **b** Avalanche breakdown electric field with different doping levels (APD-2). Thickness of charge layer is 90 nm. Red dashed lines: the field of absorption is larger than the tunneling threshold field. Black solid lines: the field of absorption is less than the tunneling threshold field. The figure description of **b** is similar to **a**. **c** Relationship between field and bias voltage in multiplication and absorption (APD-1). Thickness of charge layer is 90 nm. Electric field of multiplication (black square). Electric field of absorption (red triangle). **c** Presents the relationship between the electric field and bias voltage in multiplication and absorption layers. When the electric field in the absorption layer reaches the tunneling threshold value, the avalanche breakdown electric field in the multiplication gradually decreases. Moreover, the absorption field slope increases when the electric field in the absorption layer exceeds the tunneling threshold. **d** Relationship between field and bias voltage in multiplication and absorption (APD-2). Thickness of charge layer is 90 nm. Electric field of multiplication (black square). Electric field of absorption (red triangle). The figure legend of **d** is similar to **a**

The simulation in this part will study the tunneling effect on the electric field in the device. From the theoretical analysis, the tunneling effect has an influence on the avalanche field in multiplication. Thus, the simulation will design to study the influence of electric field in the absorption layer that exceeds the tunneling threshold value. First, group A, with the structure of APD-1, charge layer thickness of 90 nm, and different charge layer doping levels of 1.4 × 10^17^–2.4 × 10^17^ cm^−3^, was simulated for electric field distribution when the device avalanche breaks down. The result is shown in Fig. 12a. When the charge layer doping level exceeds 2.0 × 10^17^ cm^−3^, the field in the absorption layer becomes lower than the tunneling threshold field and the avalanche field in the multiplication layer reaches the same value. However, when the doping level is less than 2.0 × 10^17^ cm^−3^, the field in the absorption layer exceeds the tunneling threshold field and the avalanche field in the multiplication layer decreases with the decrease of the doping level in charge layer. Similar results were observed in the APD-2 structure (with a charge layer thickness of 90 nm and doping level of 2.2 × 10^17^–3.6*10^17^ cm^−3^) (Fig. 12b). That is, if the electric field in the absorption layer exceeds the tunneling threshold value at or over the breakdown voltage, then the breakdown electric field in the multiplication will decrease.

Groups B (APD-1 thickness of 90 nm, doping level of 2.4 × 10^17^ cm^−3^ in charge layer and APD-2 thickness of 90 nm, doping level of 3.6 × 10^17^ cm^−3^) were designed to demonstrate the relationship between the threshold electric field in the absorption layer and avalanche field in the multiplication layer. The multiplication and absorption electric fields vary with the bias voltage on the device. As shown in Fig. 12c, d, when the electric field in the absorption layer reaches the tunneling threshold value, the avalanche breakdown electric field in the multiplication gradually decreases. Moreover, when the absorption field exceeds the tunneling threshold, the avalanche breakdown electric field in the multiplication layer plummets. Furthermore, the absorption field slope increases when the electric field in the absorption layer exceeds the tunneling threshold.

The phenomenon in Fig. 12 can be explained by the theoretical analysis that tunneling has an influence on the charge density in the “Methods” section. When the electric field reaches the tunneling threshold value in the absorption layer, the charge density *ρ* becomes unequal to the dopant ion. The multiplication field will decrease as the negative ion increases, and the absorption field will increase as the positive ion increases. Concurrently, the absorption field slope will increase due to the tunneling effect. As a result, the electric field in the absorption should be less than the tunneling threshold value to maintain the high field in the multiplication layer and the low dark current when the device avalanche breaks down.

## Conclusions

In summary, we have presented a theoretical study and numerical simulation analysis involving the InGaAs/InAlAs APD. The mathematical relationship between the device parameters and electric field distribution in the device was built. And the tunneling effect was taken into consideration in the theoretical analysis. Through analysis and simulation, the influence of structure parameters on the device and the detailed relationship of each layer were fully understood in the device. Three important conclusions can be obtained from this paper. First, the doping level and thickness of the charge layer for different multiplication thicknesses can be calculated by the theoretical model in the “Methods” section. Calculated charge layer values (doping and thickness) are in agreement with the experiment results. Second, as the charge layer thickness increases, the suitable doping level range in charge layer decreases. Compared to the thinner charge layer, the performance of APD varies significantly via several percent deviations of doping concentrations in the thicker charge layer. When designing APD structures, choosing a thin charge layer will give a high level of doping tolerance, as well as confer APD with good controllability. Finally, the *G*_*btt*_ of tunneling effect was calculated, and the influence of tunneling effect on the avalanche field was analyzed. We confirm that the avalanche field and multiplication factor (*M*_*n*_) in the multiplication will decrease by the tunneling effect.

## Abbreviations

2D: Two-dimensional
APD: Avalanche photodiode
DCR: Dark count rate
SACM APDs: Separate absorption, charge, and multiplication avalanche photodiodes
SAGCMAPDs: Separate absorption, grading, charge, and multiplication avalanche photodiodes
SPAD: Single-photon avalanche photodiode
SPDE: Single-photon detection efficiency
SRH: Shockley–Read–Hall
